# Rare Coexistence of Cardiac Tuberculosis: Effusive-Constrictive Pericarditis With Right Atrial Tuberculoma and Right Atrial Myocarditis in an Immunocompetent Patient With Disseminated Tuberculosis

**DOI:** 10.7759/cureus.84119

**Published:** 2025-05-14

**Authors:** Nivy Patel, Preet Baxi, Mohammed Nadeem Patel, Mostafa Abdulrazak Ketaz Al Lami, Sagar Kabde

**Affiliations:** 1 Department of Critical Care Medicine/Cardiology, Bankers Heart Institute, Surat, IND; 2 Department of Critical Care Medicine, Bankers Heart Institute, Surat, IND; 3 Department of Cardiology, Bankers Heart Institute, Surat, IND

**Keywords:** disseminated tuberculosis, effusive-constrictive pericarditis, immunocompetent patient, right atrial myocarditis, right atrial tuberculoma

## Abstract

Cardiac involvement in tuberculosis (TB) is uncommon, and especially the concurrent occurrence of effusive-constrictive pericarditis, right atrial tuberculoma, and right atrial myocarditis is extremely rare in immunocompetent individuals. We report a case of a 22-year-old immunocompetent male with disseminated TB, presenting with this rare combination. To our knowledge, this specific coexistence has not been documented in the literature previously. The diagnosis was confirmed using 2D echocardiography, CT scan, and histopathological analysis. Echocardiography revealed pericardial effusion and signs of constrictive pericarditis, supported by respiratory variation in ventricular filling. These findings were also consistent with the patient’s clinical presentation. The right atrial tuberculoma was confirmed by histopathology of the resected tissue, and right atrial myocarditis was also proven histologically. The patient was treated with standard anti-tubercular therapy and underwent surgical resection for right atrial tuberculoma, with a favorable clinical outcome. This case also highlights the diagnostic value of histopathology when microbiological tests are inconclusive and emphasizes the need to consider TB in the differential diagnosis of cardiac masses, which can mimic various neoplastic or thrombotic lesions.

## Introduction

Cardiac involvement in tuberculosis (TB) is a rare occurrence, with pericardial disease being the most commonly reported manifestation, frequently progressing to constrictive pericarditis. In contrast, effusive-constrictive pericarditis (ECP), representing a transitional or overlapping form, has been reported in only a limited number of cases. This condition is characterized by coexisting pericardial effusion and constriction, where pericardial tamponade physiology persists despite fluid drainage due to a noncompliant visceral pericardium. Myocardial and endocardial involvement are even rarer manifestations, typically found during autopsy, with only a small number of cases reported antemortem. Myocardial TB has an estimated prevalence of 0.14%-2% [1], and endocardial tuberculomas are similarly rare, appearing in approximately 0.14% of cases and mostly identified at autopsy [2]. A tuberculoma is a localized granulomatous mass caused by *Mycobacterium tuberculosis*, typically composed of caseating necrosis and epithelioid giant cells.

We present a case of a 22-year-old immunocompetent male diagnosed with disseminated TB, involving pulmonary infiltrates, thyroid and splenic nodules, and mediastinal lymphadenopathy. Notably, this patient also exhibited a coexistence of three rare cardiac findings: rapidly progressive and symptomatic ECP, an endocardial tuberculoma located in the right atrium, and right atrial myocarditis. Although pericardial involvement in cardiac TB is well-documented in the literature, the simultaneous occurrence of these three distinct cardiac manifestations in an immunocompetent host constitutes an exceptionally rare clinical scenario, not previously reported to our knowledge.

## Case presentation

A 22-year-old immunocompetent male patient was admitted to our hospital with progressive dyspnea, cough with expectoration, loss of appetite, and significant weight loss over the past eight months, having lost approximately 15 kg during this period. He reported four to five episodes of low-grade fever during the same timeframe, which responded to antipyretics and antibiotics prescribed by a peripheral rural hospital; however, no investigations were performed at the time. His past medical history was unremarkable for diabetes mellitus, HIV infection, or other immunosuppressive conditions. Bacillus Calmette-Guérin (BCG) vaccination records were unavailable, and no vaccination scar was observed.

**Figure 1:**
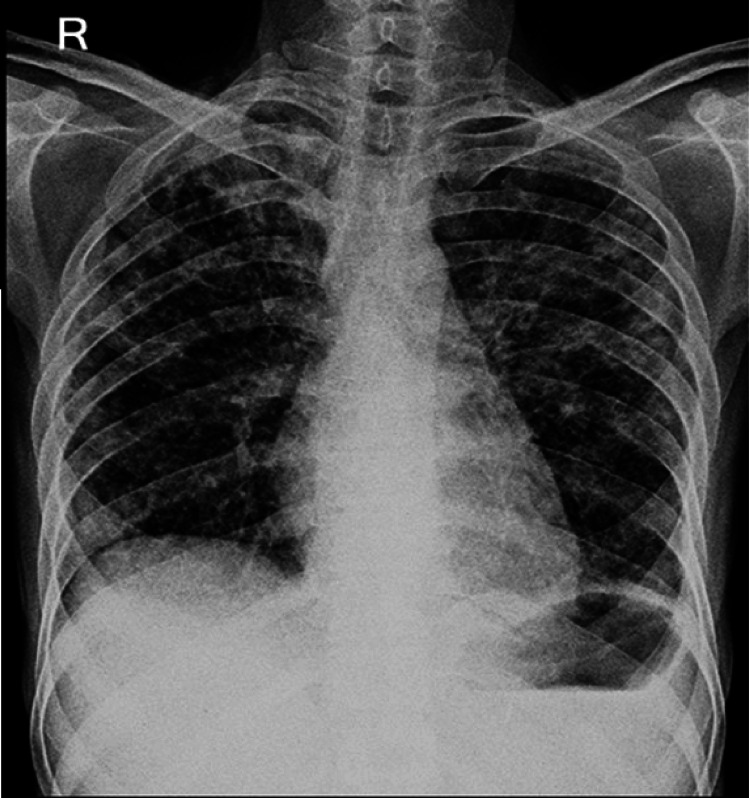
Chest X-ray (posteroanterior view) showing bilateral multiple reticulonodular infiltrates in the upper, mid, and lower zones.

On clinical examination, the patient had tachycardia, elevated jugular venous pressure (JVP), and supraclavicular and axillary lymphadenopathy. Auscultation revealed a pericardial rub over the apical area and coarse crepitations in all lung fields. Chest radiography demonstrated bilateral multiple reticulonodular infiltrates in the upper, mid, and lower lung zones (Figure 1).

Laboratory investigations revealed anemia (hemoglobin: 8.4 g/dL) and leukocytosis (13,600/mm³). Liver and renal function tests were within normal limits, while C-reactive protein (CRP) and erythrocyte sedimentation rate (ESR) were significantly elevated (55.7 mg/L and 155 mm/hr, respectively) (Table 1). HIV enzyme-linked immunosorbent assay (ELISA) was negative. Three sputum samples were negative for acid-fast bacilli (AFB), but the GeneXpert MTB/RIF (*Mycobacterium tuberculosis*/rifampicin) assay (C4) detected *Mycobacterium tuberculosis* at a very low level.

**Table 1:** Laboratory investigations. MCV: mean corpuscular volume; MCH: mean corpuscular hemoglobin; MCHC: mean corpuscular hemoglobin concentration; RDW: red cell distribution width; CRP: C-reactive protein; ESR: erythrocyte sedimentation rate; SGPT: serum glutamic pyruvic transaminase; ALT: alanine aminotransferase; SGOT: serum glutamic oxaloacetic transaminase.

Hematology - complete blood count			
				Parameter	Result	Unit	Fixed range text
Hemoglobin (HGB)	8.4	gm%	11 - 15
Hematocrit (HCT)	24.6	%	36 - 48
Red blood cell (RBC) count	2.95	million/cu.mm	3.50 - 5.50
MCV	83.4	fL	76 - 96
MCH	27.8	pg	27 - 31
MCHC	33.3	gm/dl	32 - 36
RDW (SD)	15.8	%	12 - 16
Total WBC count	13,600	cell/cu.mm	4000 - 10000
Platelet count	265	Thou/cu.mm	150.0 - 410.0
Platelets on smear	Adequate		
CRP	55.7	mg/L	<10
ESR	155	mm/hr	0 - 15
Biochemistry			
Parameter	Result	Unit	Fixed range text
S. creatinine	0.8	mg/dl	0.60 - 1.10
SGPT (ALT)	19.4	IU/L	0 - 49
SGOT	20.6	U/L	8 - 45
Total proteins	6.8	gm/dl	6.0 - 8.3
Albumin	3.5	gm/dl	3.5 - 5.5

**Figure 2:**
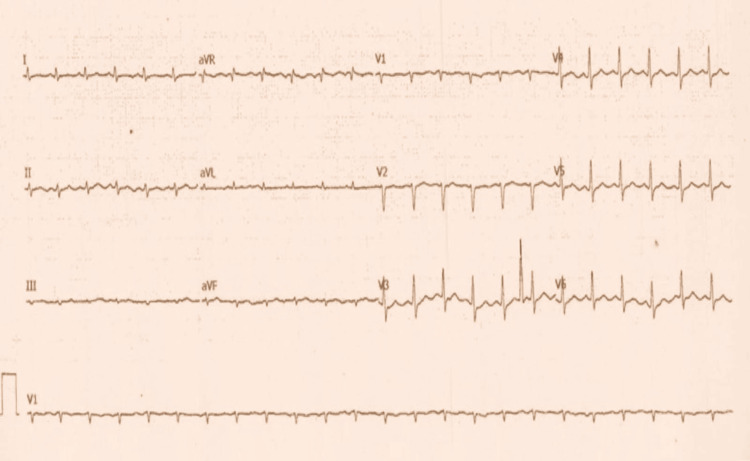
ECG on admission showing low voltage complexes with sinus tachycardia.

**Figure 3:**
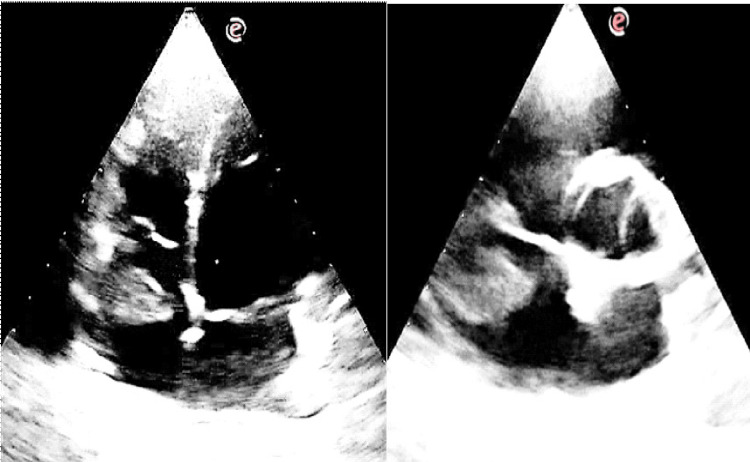
Two-dimensional echocardiography apical four-chamber view showing echogenic mass in the right atrial (RA) attached to the free wall (left) and modified parasternal short axis view showing mass in the RA (right).

**Figure 4:**
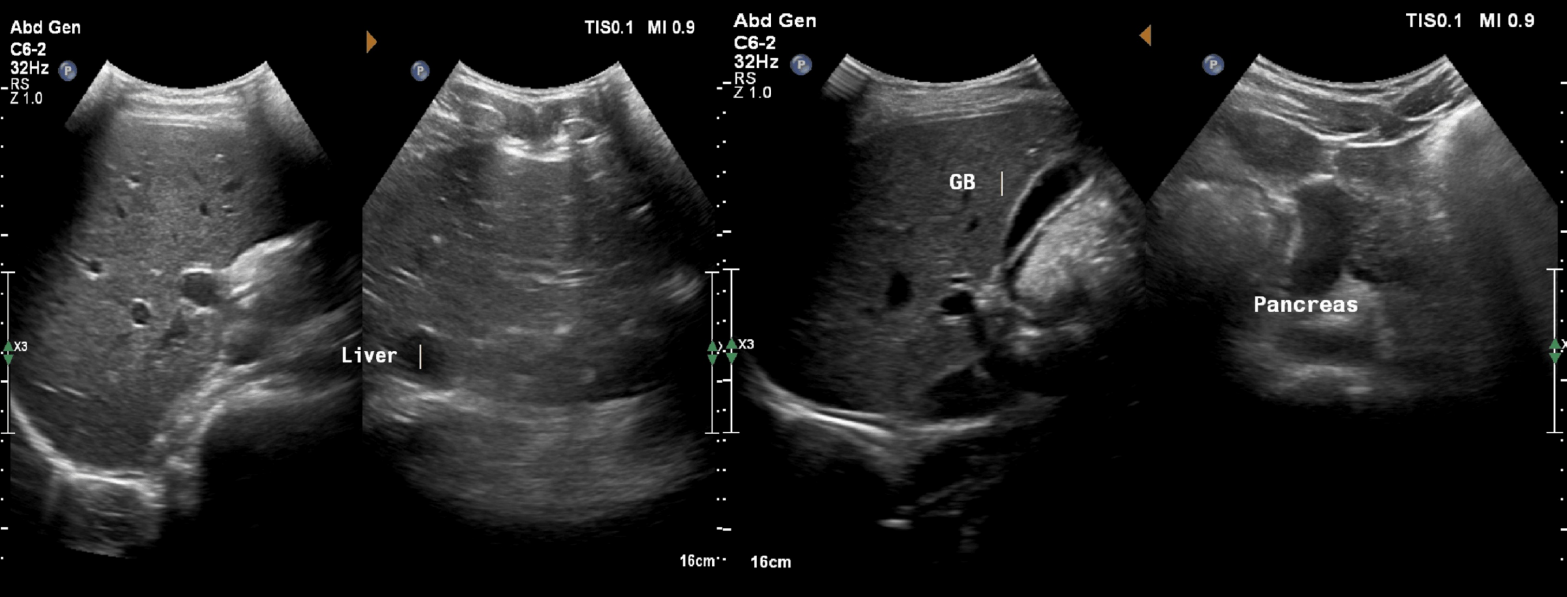
Abdominal ultrasonography was unremarkable.

Given the presence of elevated JVP and a pericardial rub, an electrocardiogram (ECG) and two-dimensional echocardiography (2D Echo) were performed. ECG showed sinus tachycardia (Figure 2), while echocardiography revealed a 2.5 mm × 3.0 mm mass in the right atrium, attached to the free wall (Figure 3). The mass did not extend into the superior vena cava (SVC) or inferior vena cava (IVC) and did not involve the tricuspid valve. Additionally, pericardial thickening with mild pericardial effusion was noted, but there were no signs of constrictive pericarditis. Abdominal ultrasonography (USG) was unremarkable (Figure 4).

The patient was initiated on anti-tubercular therapy (ATT) as per national TB guidelines. Given the pericardial involvement, he was also started on prednisolone (1 mg/kg) for four weeks, followed by a gradual taper over the subsequent four weeks, to prevent constrictive pericarditis. However, the nature of the right atrial (RA) mass remained uncertain. A differential diagnosis including thrombus, myxoma, carcinoid tumor, and tuberculous granuloma was considered. As right atrial thrombi have been reported in various conditions, a trial of anticoagulation therapy was given for four weeks, but the mass size remained unchanged. After initiating ATT, the patient showed symptomatic improvement and was subsequently discharged with regular follow-up.

**Figure 5:**
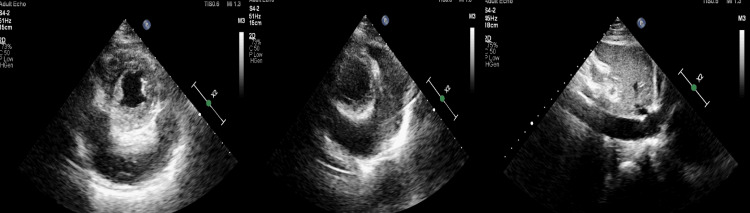
Pericardial effusion with inferior vena cava dilation.

At the two-month follow-up, he reported worsening dyspnea over the past 15 days. Clinical examination revealed tachycardia, elevated JVP, hypotension, muffled heart sounds, mild ascites, and pedal edema. Subsequently, transthoracic echocardiography showed signs of cardiac tamponade, with right ventricular collapse along with IVC dilation (Figure 5). These findings were highly suggestive of impaired diastolic filling and tamponade physiology, present along with persistent right atrial mass. The patient was immediately admitted, and emergency pericardiocentesis was performed.

**Figure 6:**
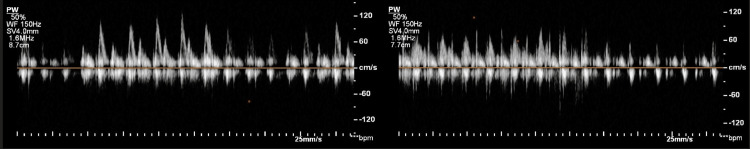
Change in blood flow velocity across mitral (left) and tricuspid (right) valves during different phases of respiration.

**Figure 7:**
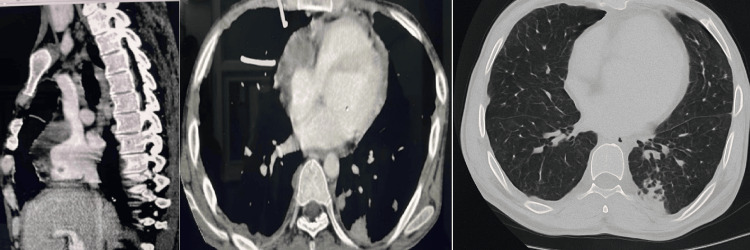
CT of the thorax showing diffuse non-enhancing thickening along the anterior wall of the right atrial (RA) and along the anterior aspect (left). Mass seen in the right atrium along with a thickened RA wall and pericardial thickening (center), with bilateral reticulonodular infiltration (right).

Post-procedure echocardiography revealed only a minimal residual pericardial effusion. Over the following two days, the patient reported some improvement in dyspnea; however, he continued to experience symptoms even with minimal exertion (New York Heart Association (NYHA) class III). A repeat echocardiogram was performed at the same time, which revealed obvious signs of constrictive pericarditis. These included septal bounce, along with exaggerated ventricular interdependence and respiratory variation in mitral and tricuspid blood flow velocities, characteristic of pericardial constriction (Figure 6). Furthermore, CT of the thorax demonstrated a thickened pericardium (>2 mm), predominantly on the RA side. Based on this, a diagnosis of ECP was made. There was diffuse, non-enhancing thickening along the anterior RA wall and anterior aspect of the SVC, with maximum thickening measuring 18 mm and extending 88 mm superoinferiorly. A focal, round, non-enhancing lesion (2.8 mm × 2.9 mm) was attached to the thickened RA. Adjacent pleural thickening, mild bilateral pleural effusion, mediastinal lymphadenopathy, and nodules in the right thyroid lobe and spleen were also noted (Figure 7). MRI was not done as it was unlikely to help identify the cause of the condition, and the cost was also a limiting factor.

Given the NYHA class III dyspnea, classic signs of constrictive pericarditis, failed steroid therapy, and persistent RA mass with RA infiltration, surgical intervention was planned to remove the RA mass and relieve pericardial constriction.

Subtotal (anterior) pericardiectomy was performed, and the gross specimen of the RA mass was a whitish-brown, fragile tissue, with multiple yellowish tubercles on the pericardium. The average pericardial thickness was approximately 5 mm. Specimens from the RA mass, pericardium, and RA infiltration were sent for histopathological examination.

**Figure 8:**
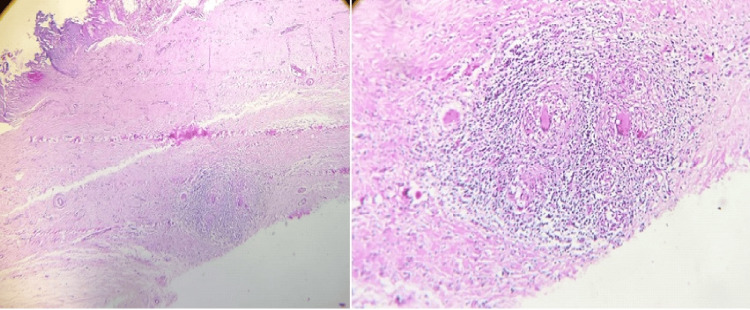
Histology of the pericardium showing granulomatous inflammation and caseous necrosis (left), and right atrial mass shows granulomatous inflammation with multinuclear giant cells (right).

Frozen section histopathological examination of the right atrial mass revealed extensive caseous necrosis with granulomatous inflammation. The granulomas were composed of epithelioid cells and multinucleated giant cells, surrounded by fibrinous amorphous material, suggestive of an organizing thrombus. Pericardial biopsy demonstrated multifocal granulomatous inflammation with central caseation and associated fibrosis, consistent with tuberculous pericarditis (Figure 8). Histopathological analysis of the right atrial myocardium revealed definitive features of granulomatous myocarditis, characterized by dense lymphocytic infiltrates, caseating granulomas, and the presence of Langhans-type giant cells. Intraoperative findings reported by the cardiothoracic surgical team included a markedly thickened right atrial pericardium. Left ventricular function was preserved as well. These findings supported a diagnosis of right atrial myocarditis. Ziehl-Neelsen staining for AFB for pericardial fluid and tissue was negative, although this does not exclude a tuberculous etiology, given the characteristic histological features.

Based on the patient’s prior history of TB and histopathological findings, a final diagnosis of tuberculous ECP, accompanied by a right atrial endocardial tuberculoma and myocarditis of the right atrium, was established. The patient was continued on ATT.

At the one-month follow-up, the patient showed significant clinical improvement, with resolution of symptoms and weight gain. Echocardiography showed no residual right atrial mass, with only mild tricuspid regurgitation and normal pulmonary artery pressure.

## Discussion

Pericardial involvement is relatively more frequent in patients with cardiac TB and may present as acute pericarditis, pericardial effusion, myopericarditis, or constrictive pericarditis. On the other hand, ECP is an infrequent pericardial disease that can be overlooked in patients initially presenting with cardiac tamponade. While progression to chronic constrictive pericarditis is commonly observed and occurs over several months, spontaneous resolution has been reported, particularly in idiopathic cases [3]. On the contrary, this case shows a rapid progression from pulmonary TB to pericarditis and subsequent effusion and constriction, accompanied by a right atrial tuberculoma. The progression to constrictive pericarditis, despite corticosteroid treatment, highlights the issue of delayed diagnosis and treatment. Before the initial presentation, the patient's fever was managed symptomatically without identifying the underlying cause, leading to untreated TB and the subsequent development of extensive fibrotic and granulomatous changes in the pericardium. This delay in addressing the root cause could have led to rapid progression of the disease.

ECP has a broad spectrum of underlying causes, including iatrogenic factors such as prior cardiac surgery or thoracic irradiation, as well as chest trauma and infectious agents. Among the infectious causes, mycobacterial species are commonly associated with pericarditis in immunocompromised patients. In contrast, their involvement in immunocompetent individuals is rare and has been reported only anecdotally [4].

TB has a predilection for right-sided mediastinal lymph nodes, making the right heart, particularly the free wall of the right atrium, highly susceptible through contiguous spread [5]. This is consistent with our case, where the right atrial free wall of the endocardium was involved, in the form of a tuberculoma. Furthermore, tuberculoma may infiltrate the underlying myocardium, causing ulceration of the surface, leading to thrombus formation and subsequent thromboembolism. This phenomenon may give rise to hematogenous seeding and disseminated TB, as was seen in this case [5]. The final histology confirmed tuberculoma despite the presence of organizing thrombus elements.

Myocardial involvement in TB is exceedingly rare and is diagnosed on autopsy [2]. In a case series by Custer and Charr, an analysis of over 14,000 TB-related deaths revealed cardiac TB in only 64 instances (0.5%) [6]. The infection may reach the myocardium via hematogenous dissemination, retrograde lymphatic spread from mediastinal lymph nodes, or direct extension from the pericardium.

The differential diagnosis of a right atrial mass includes thrombus, right atrial myxoma, lymphoma, myosarcoma, rhabdomyosarcoma, vascular tumors, and metastatic lesions. A well-defined mass originating from the right atrial free wall with myocardial infiltration typically raises suspicion for a cardiac tumor or thrombus rather than a tuberculoma. Consequently, in our patient, tuberculoma was not initially considered. Surgical treatment of cardiac tuberculoma is typically considered only when the diagnosis is uncertain, when there is inadequate response to antitubercular therapy, or if serious cardiac complications develop [7]. In this case, due to persistent mass and severely symptomatic constrictive pericarditis despite ATT pharmacotherapy, surgery was performed.

Gonzalez et al. [8] reported an HIV-positive patient with disseminated TB and ECP. Despite urgent pericardiocentesis, the patient deteriorated and died before pericardiectomy could be performed. Haq et al. [9] described a case of effusive-constrictive TB pericarditis with biventricular dysfunction, which improved with steroids and anti-TB therapy. Sabzi et al. [10] reported a right atrial tuberculoma enclosed by thrombus. The mass was easily dissected from the interatrial septum and excised through a median sternotomy.

## Conclusions

Clinicians should maintain a strong suspicion for TB even in immunocompetent patients with unexplained cardiac masses or pericardial manifestations, especially in endemic areas. This case further underscores the diagnostic utility of histopathology in confirming myocardial and endocardial TB, as limitations persist in the usage of imaging alone to confirm diagnosis, and the therapeutic role of early surgical intervention when there is persistent mass or constriction despite medical therapy.

Disseminated TB in immunocompetent individuals is uncommon, and cardiac involvement beyond the pericardium is particularly rare. Right atrial masses are encountered in clinical practice, but tuberculous involvement of the right atrium is exceedingly rare. In conclusion, the simultaneous presence of rapidly deteriorating and symptomatic ECP with right atrial tuberculoma and right atrial myocarditis in this immunocompetent patient represents a rare combination of findings, with only a few similar cases described in the literature.
